# Meglumine acridone acetate, the ionic salt of CMA and *N*-methylglucamine, induces apoptosis in human PBMCs via the mitochondrial pathway

**DOI:** 10.1038/s41598-019-54208-9

**Published:** 2019-12-03

**Authors:** Marina A. Plotnikova, Sergey A. Klotchenko, Artem A. Kiselev, Andrey N. Gorshkov, Anna-Polina S. Shurygina, Kirill A. Vasilyev, Urszula Uciechowska-Kaczmarzyk, Sergey A. Samsonov, Alexey L. Kovalenko, Andrey V. Vasin

**Affiliations:** 1 Smorodintsev Research Institute of Influenza, St. Petersburg, Russia; 2 Almazov National Medical Research Centre, St. Petersburg, Russia; 3 Faculty of Chemistry, University of Gdańsk, Gdańsk, Poland; 4 Institute of Toxicology, Federal Medical-Biological Agency of Russia, St. Petersburg, Russia; 5 Institute of Biomedical Systems and Botechnologies, Peter the Great St. Petersburg Polytechnic University, St. Petersburg, Russia; 6 Saint Petersburg State Chemical Pharmaceutical University, St. Petersburg, Russia

**Keywords:** Virtual drug screening, Drug screening, Computer modelling

## Abstract

Meglumine acridone acetate (MA) is used in Russia for the treatment of influenza and other acute respiratory viral infections. It was assumed, until recently, that its antiviral effect was associated with its potential ability to induce type I interferon. Advanced studies, however, have shown the failure of 10-carboxymethyl-9-acridanone (CMA) to activate human STING. As such, MA’s antiviral properties are still undergoing clarification. To gain insight into MA’s mechanisms of action, we carried out RNA-sequencing analysis of global transcriptomes in MA-treated (MA+) human peripheral blood mononuclear cells (PBMCs). In response to treatment, approximately 1,223 genes were found to be differentially expressed, among which 464 and 759 were identified as either up- or down-regulated, respectively. To clarify the cellular and molecular processes taking place in MA+ cells, we performed a functional analysis of those genes. We have shown that evident MA subcellular localizations are: at the nuclear envelope; inside the nucleus; and diffusely in perinuclear cytoplasm. Postulating that MA may be a nuclear receptor agonist, we carried out docking simulations with PPARα and RORα ligand binding domains including prediction and molecular dynamics-based analysis of potential MA binding poses. Finally, we confirmed that MA treatment enhanced nuclear apoptosis in human PBMCs. The research presented here, in our view, indicates that: (i) MA activity is mediated by nuclear receptors; (ii) MA is a possible PPARα and/or RORα agonist; (iii) MA has an immunosuppressive effect; and (iv) MA induces apoptosis through the mitochondrial signaling pathway.

## Introduction

**Figure 1:**
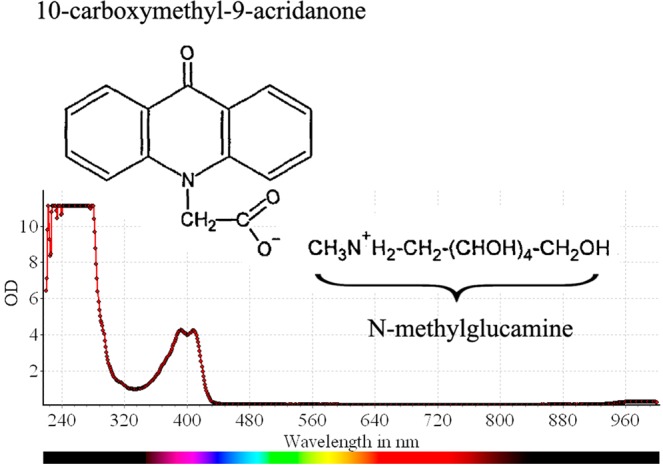
Chemical structure of N-methylglucamine acridone acetate (MA; C_22_H_28_N_2_O_8_). The 10-carboxymethyl-9-acridanone (CMA) anion and the N-methylglucamine cation are shown. The MA absorption spectrum is presented at the bottom.

N-methylglucamine acridone acetate (MA) is the active ingredient in the pharmaceutical drug Cycloferon which is intended for use as an immunomodulator in the treatment of infectious diseases^1^. MA is the ionic salt of 10-carboxymethyl-9-acridanone (CMA) and *N*-methylglucamine (Fig. 1). In 1976, Kramer *et al*. demonstrated the activity of CMA sodium salt against both RNA and DNA viruses in mice^2^. The therapeutic effect of CMA has been described as interferon-like. Later, it was shown that CMA possesses interferon inducing activity and that such activity occurs at the level of type I interferon genes’ mRNA transcription, not at the level of post-transcriptional processes^3^.

A number of studies have noted the species-specific effects of CMA. In particular, the induction of endogenous interferon by CMA has been shown in mice and in murine cell cultures, but not in human or rabbit cells^2–4^. In 2013, Cavlar showed that stimulation of type I interferon production occurs through binding of CMA to the endoplasmic reticulum protein STING^5^. The ligand-binding domains of the human and murine STING proteins are structurally different, and this fact causes the proteins to have different affinities for CMA^5,6^. Those findings explain why the ability of CMA to induce interferon synthesis is different in mouse and human cells.

Pharmaceuticals based on CMA or MA, in particular, are widely used in Russia and in the Commonwealth of Independent States. The antiviral activity of MA has been established using both *in vitro* and *in vivo* methods. These studies have included experimental models of influenza and herpes virus infection as well as models of the immunodeficiency virus, hepatitis C and tick-borne encephalitis^7,8^. Anti-tumor effects have also been shown with MA. This effect was seen as a strengthening of the therapeutic effect of chemotherapy drugs^9^. MA has been shown to have restorative effects in mucosa^7,10^. The effect is mediated by gamma interferon synthesis during the repair and regeneration processes that occur in damaged epithelium. While the immuno-modulatory effects of MA have been shown in experimental models and in clinical practice^7^, little is known, however, about MA’s mechanism of action. In this study, an evaluation of CMA’s biological activity and pharmacological potential is presented. Effects were shown using transcriptome profiling of human peripheral blood mononuclear cells (PBMCs).

## Results

### Sequencing and statistical analysis of data

Ribonucleic acid sequencing (RNA-Seq) was used to evaluate gene expression in four groups of samples: (1) mock PBMCs (MA^−^Inf^−^ controls); (2) PBMCs infected with the influenza А/California/07/09 (H1N1)pdm09 strain (MA^−^Inf^−^); (3) PBMCs stimulated by MA (MA^+^Inf^−^); and (4) PBMCs infected with the influenza А/California/07/09 (H1N1)pdm09 strain and also stimulated by MA (MA^+^Inf^+^). Each group was represented by two biological samples. Approximately 2–3 million reads per library were produced, and 76–79% of these reads have been successfully mapped to the GRCh38 human genome assembly in a paired-end (2 × 75 bp) mode (see Supplementary Table S2).

**Figure 2:**
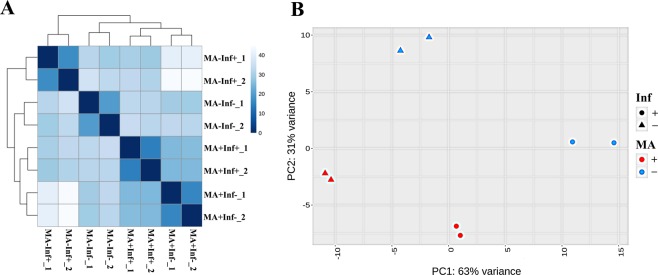
Comparison of differentially expressed genes in MA-treated and untreated PBMCs. (**A**) Heatmap of Euclidean sample distances after regularized-logarithm transformation of RNA-Seq data; (**B**) PCA of samples after rlog transformation.

In order to assess overall similarity between samples in the RNA sequencing data, we calculated the Euclidean distances between samples and visualized them as a heatmap (Fig. 2A). We also performed principal component analysis (PCA) to assess whether gene expression revealed transcription profiles specific for MA-treated or untreated cells (Fig. 2B). According to the heatmap, PCA showed a clear distinction between genes expressed in all four groups, depending on sample type.

**Figure 3:**
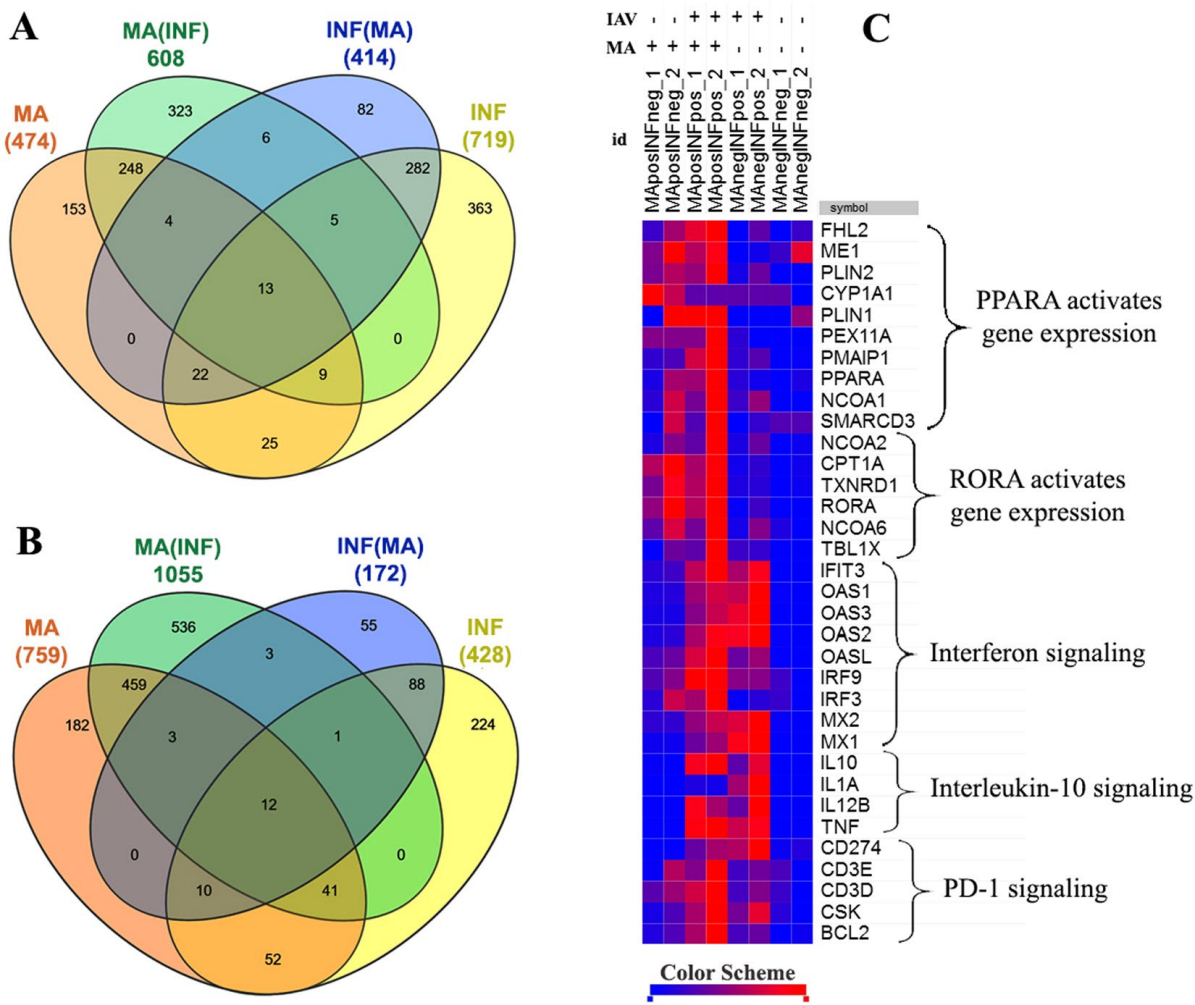
Venn diagram of differentially expressed (DE) genes. The intersection of up-regulated (**A**) and down-regulated (**B**) DE genes among four expression datasets: MA^+^Inf^−^ vs MA^−^Inf^−^ (MA); MA^+^Inf^+^ vs MA^−^Inf^+^ (MA(Inf)); MA^−^Inf^+^ vs MA^−^Inf^−^ (Inf:) and MA^+^Inf^+^ vs MA^+^Inf^−^ (Inf(MA). Comparisons were executed for DE genes with STAT >0 (for UP) or <0 (for DOWN), and *adjusted p*-value  < 0.05. Overall heatmap the DE genes (**C**).

For the purpose of analyzing differential gene expression, experimental groups were paired with their controls based on the presence or absence of MA treatment. These comparisons (MA^+^Inf^−^ compared with MA^−^Inf^−^; and MA^+^Inf^+^ compared with MA^−^Inf^+^) featured a cutoff limit of log_2_(fold change) >2 and an *adjusted p*-value < 0.5. In that manner, we identified 459 down-regulated and 248 up-regulated differentially expressed genes, specific to MA, in both infected and virus free PBMCs (Fig. 3A,B). As shown in Fig. 3C, clustering of the differentially expressed genes, restricted to log2(fold change) (<−1 or >+1) and *p*-value (<0.005), shows clearly distinguishable gene patterns across the biological pathways.

According to the data, treatment of cells with MA led to the suppressed expression of a significant number of genes (759). At the same time, only about 474 genes showed increased expression in response to MA.

### Functional analysis of differentially expressed genes

In order to gain some understanding of the molecular and cellular processes taking place in MA stimulated cells, we performed a functional analysis of differentially expressed genes using the Reactome database^11^.

When cells were treated with MA, both in the presence or absence of influenza virus infection, mRNAs of genes involved in nuclear receptor signaling (thyroid hormone receptor-like subfamily) were activated (in human PBMCs). In particular, a number of genes from the metabolism of retinoic acid, the Retinoic Acid Receptor-related Orphan Receptor alpha (RORα), and Peroxisome Proliferator-Activated Receptor alpha (PPARα) pathways showed increased expression, such as CPT1A, TXNRD1, and PDK4.

PPARs are lipid-activated transcription factors which regulate mitochondrial and peroxisomal β-oxidation activities^12^. RORα is involved in the metabolism of cholesterol and triglycerides, the regulation of circadian rhythm, and in immune function, such as the differentiation of T helper 17 cells (T_h_17)^13,14^. Along with activation of nuclear receptors, MA induced expression of pro-apoptotic genes (PMAIP1) in PBMCs. Metabolic pathways were activated in which pro-apoptotic members of the Bcl-2 protein family, such as Noxa and BH3-only protein, participate. The pathway connected with release of cytochrome c to the cytoplasm was also activated. Most likely, MA-induced apoptosis is mediated through the intrinsic apoptosis pathway (*p*-value = 4.32E-03). In other words, it likely occurs via mitochondrial factors, not by the activation of so-called “death receptors”.

MA caused PBMCs to increase the expression of IL24 mRNA, a cytokine belonging to the IL10 superfamily. The selective induction of apoptosis under the influence of IL24 has been confirmed many times^15,16^. Specifically, the MDA-7/IL-24 protein promotes activation of the p38 Mitogen Activated Protein Kinase (MAPK) pathway. GADD (growth arrest and DNA damage) promotes growth arrest and cell death in the pathway^17,18^. We found that in MA-stimulated cells, GADD45A levels were also elevated in the presence of viral infection.

It was found that, when cells are treated with MA, CR1 (CD35) mRNA expression was increased. CR1 interacts with RUNX1 and FOXP3, which control development of the regulatory T lymphocyte pathway (*p*-value = 0.003). CR1 is a multifunctional glycoprotein that has complement regulatory and anti-inflammatory activities^19,20^. Studies, using various cell lines, have shown that retinoic acid up-regulates CR1 gene expression^21^. Treatment of PBMCs with MA (in the absence of infection) led to the induction of IFIT2, IFIT3, and OAS genes; they are associated with categories Interferon alpha/beta Signaling (*p*-value = 7.25E-4) and Interferon Signaling (*p*-value = 7.76E-3) (Table 1). However, noteworthy increases in the expression of mRNAs associated with other regulators of cellular or humoral immunity were not seen.

**Table 1:** Metabolic pathways activated in PBMCs under the influence of MA.

Pathway identifier in Reactome	Pathway Name	Entities				Reactions	
		Found	Ratio	*p*-value	FDR	Found	Ratio
R-HSA-111448	Activation of NOXA and translocation to mitochondria	2/6	4.29E-04	5.83E-04	6.75E-02	5/5	4.2E-04
R-HSA-909733	Interferon alpha/beta signaling	6/184	1.30E-02	7.25E-04	6.75E-02	2/20	2.0E-03
R-HSA-1475029	Reversible hydration of carbon dioxide	2/17	1.00E-03	4.49E-03	2.02E-01	2/8	6.8E-04
R-HSA-5339562	Uptake and actions of bacterial toxins	3/60	4.00E-03	5.25E-03	2.02E-01	7/65	6.0E-03
R-HSA-419408	Lysosphingolipid and LPA receptors	2/19	1.00E-03	5.57E-03	2.02E-01	1/3	2.5E-04
R-HSA-5210891	Uptake and function of anthrax toxins	2/22	2.00E-03	7.39E-03	2.02E-01	6/19	2.0E-03
R-HSA-913531	Interferon Signaling	7/392	2.80E-02	7.76E-03	2.02E-01	14/66	6.0E-03
R-HSA-1368082	RORα activates gene expression	2/25	2.00E-03	9.43E-03	2.17E-01	2/4	3.4E-04
R-HSA-6803204	TP53 Regulates Transcription of Genes Involved in Cytochrome C Release	2/33	2.00E-03	1.60E-02	2.91E-01	2/25	2.0E-03
R-HSA-1989781	PPARα activates gene expression	4/174	1.20E-02	1.87E-02	2.91E-01	2/41	3.0E-03
R-HSA-114452	Activation of BH3-only proteins	2/36	3.00E-03	1.88E-02	2.91E-01	5/19	2.0E-03
R-HSA-400206	Regulation of lipid metabolism by Peroxisome proliferator-activated receptor alpha (PPARα)	4/176	1.30E-02	1.94E-02	2.91E-01	2/44	4.0E-03
R-HSA-5578999	Defective GCLC causes Hemolytic anemia due to gamma-glutamylcysteine synthetase deficiency (HAGGSD)	1/4	2.86E-04	2.29E-02	3.21E-01	1/1	8.5E-05
R-HSA-109606	Intrinsic Pathway for Apoptosis	2/52	4.00E-03	3.69E-02	4.17E-01	7/47	4.0E-03

Both uninfected and mock cells were studied. Pathways with Entities *p-*value < 0.05 are shown.

In contrast, there was significant suppression (Table 2) of mRNA from genes involved in IL-10 signaling (*p*-value = 2E-15), in signaling by interleukins IL-4 and IL-13 (*p*-value = 7.65E-9), as well as in those from interferon gamma signaling pathways (*p*-value = 1.11E-16). The main function of IL-10 is the induction of an anti-inflammatory response mediated through the IL-10 receptor^22^. IL-10 signaling exerts a number of effects. It inhibits the expression of MHC class II (HLA), as well as the expression of the surface co-stimulatory molecules CD80/CD86; signaling also reduces the production of pro-inflammatory cytokines^23,24^.

**Table 2:** Metabolic pathways suppressed in PBMCs under the influence of MA.

Pathway identifier in Reactome	Pathway Name	Entities				Reactions	
		Found	Ratio	*p*-value	FDR	Found	Ratio
R-HSA-202430	Translocation of ZAP-70 to Immunological synapse	21/42	0.003	1.11E-16	1.23E-14	4/4	3.4E-04
R-HSA-389948	PD-1 signaling	22/45	0.003	1.11E-16	1.23E-14	4/4	3.4E-04
R-HSA-202427	Phosphorylation of CD3 and TCR zeta chains	21/45	0.003	1.11E-16	1.23E-14	5/7	5.9E-04
R-HSA-877300	Interferon gamma signaling	40/250	0.018	1.11E-16	1.23E-14	4/15	1.0E-03
R-HSA-168256	Immune System	183/2641	0.189	1.11E-16	1.23E-14	395/1493	1.3E-01
R-HSA-1280215	Cytokine Signaling in Immune system	96/1055	0.075	1.11E-16	1.23E-14	94/639	5.4E-02
R-HSA-202433	Generation of second messenger molecules	21/58	0.004	7.77E-16	7.38E-14	3/17	1.0E-03
R-HSA-6798695	Neutrophil degranulation	54/480	0.034	1.67E-15	1.38E-13	10/10	8.5E-04
R-HSA-6783783	Interleukin-10 signaling	24/86	0.006	2.00E-15	1.48E-13	3/15	1.0E-03
R-HSA-2132295	MHC class II antigen presentation	28/148	0.011	1.21E-13	7.96E-12	25/26	2.0E-03
R-HSA-388841	Costimulation by the CD28 family	23/97	0.007	2.17E-13	1.30E-11	17/34	3.0E-03
R-HSA-913531	Interferon Signaling	44/392	0.028	9.03E-13	4.97E-11	8/66	6.0E-03
R-HSA-449147	Signaling by Interleukins	54/640	0.046	1.03E-10	5.26E-09	74/491	4.2E-02
R-HSA-168249	Innate Immune System	83/1302	0.093	7.68E-10	3.61E-08	212/651	5.5E-02
R-HSA-202424	Downstream TCR signaling	21/124	0.009	1.02E-09	4.50E-08	1/24	2.0E-03
R-HSA-6785807	Interleukin-4 and Interleukin-13 signaling	26/211	0.015	7.65E-09	3.14E-07	10/46	4.0E-03
R-HSA-202403	TCR signaling	21/146	0.01	1.68E-08	6.53E-07	13/52	4.0E-03
R-HSA-8957275	Post-translational protein phosphorylation	16/109	0.008	6.53E-07	2.42E-05	1/1	8.5E-05
R-HSA-1280218	Adaptive Immune System	59/997	0.071	3.33E-06	1.17E-04	90/261	2.2E-02
R-HSA-381426	Regulation of Insulin-like Growth Factor (IGF) transport and uptake by Insulin-like Growth Factor Binding Proteins (IGFBPs)	16/127	0.009	4.50E-06	1.49E-04	1/14	1.0E-03

.Both uninfected and mock cells were studied. Pathways with Entities *p*-value < 5.00E^-05^ are shown.

It has been shown that MA treated cells have reduced mRNA levels for CD86, ICAM1, IL1b, HLA D genes belonging to class II (HLA-DPA1, HLA-DRA, HLA-DRB1, HLA-DRB5), and others. The Th2 cytokines IL4 and IL13 regulate “alternative activation” of macrophages, inducing an anti-inflammatory phenotype^25,26^.

In addition, in both normal and in influenza infected PBMCs, MA suppressed mRNA levels of genes involved in T-lymphocyte activation. T cell activation is a complex process that involves at least two signals. The primary signal is delivered by the T-Cell Receptor (TCR) when it recognizes its specific antigen presented in association with the major histocompatibility complex; there is also an additional nonspecific signal mediated by cell surface receptor interactions^27^. Under the influence of MA, the involvement of several cellular pathways was seen, such as antigen-specific TCR signaling (downstream TCR signaling; translocation of ZAP-70 to immunological synapse; MHC class II antigen presentation pathways) and signaling by the CD28 costimulatory receptor (costimulation by the CD28 family and PD-1 signaling pathways).

The key role in the suppression of T cell inflammatory activity is played by Programmed Cell Death Protein 1 (PD-1), which is a member of the CD28 superfamily of immune receptors (negative regulators of TCR signaling)^28,29^. The binding of PD-L1 to PD-1 transmits an inhibitory signal that affects T cell functions. Reduced IFNγ production, limited proliferation and increased T cell apoptosis are observed^30,31^. Differential expression analysis of MA treated cells (both normal and infected) showed that expression of Programmed Death-Ligand 1 (PD-L1) was reduced.

It is interesting to note that, in response to MA, Suppressor Of Cytokine Signaling (SOCS) family members showed interesting differential expression reactions. SOCS2 mRNA was reduced both in influenza-infected (MA^+^Inf^+^) and in non-infected cells (MA^+^Inf^−^). Reduction in SOCS3 mRNA was only seen in the absence of influenza infection (MA^+^Inf^−^). At low levels, SOCS2 inhibits several signaling cascades such as the growth hormone prolactin and interleukins. At high levels, however, SOCS2 restores or even increases responsiveness to these growth factors^31–33^. Treatment of cells with MA caused a reduction in expression of the Cytochrome P450 Sterol 27-hydroxylase gene (CYP27A1). This enzyme is expressed in the mitochondria of many tissues.

**Figure 4:**
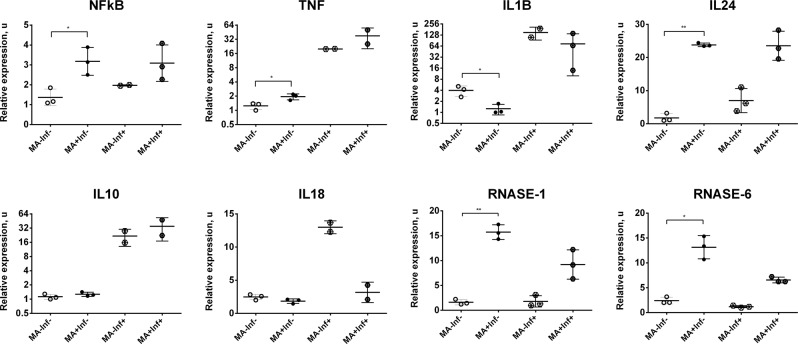
Expression profile changes in MA-treated cells including untreated, infected, and combined controls. Data are represented as mean ± SD. *p-value < 0.05, **p-value < 0.001.

CYP27 catalyzes the first reaction of bile acid (steroid acids) biosynthesis via the acidic pathway^34,35^. In response to stimulation (PBMCs + MA), the mitochondrial protein Manganese Superoxide Dismutase (MnSOD or SOD2) also showed reduced mRNA expression. SOD2 reduces the amount of mitochondrial reactive oxygen species (ROS) and, as a result, plays an anti-apoptotic role during oxidative stress, ionizing radiation, or inflammatory cytokine presence^36,37^. The expression of a subset of differentially expressed (DE) genes was validated by qRT-PCR (Fig. 4).

### Study of MA intracellular localization

MA shows the ability to fluoresce like a number of other compounds in the acridonic series. We measured the absorption and emission spectra of MA (Fig. 1). The emission spectrum was observed in the 430–460 nm range, which coincides with previously published data^7,38^. MA’s spectral properties permitted study of its localization and dynamics in living cells (*in vitro*) using confocal laser scanning microscopy (CLSM).

**Figure 5:**
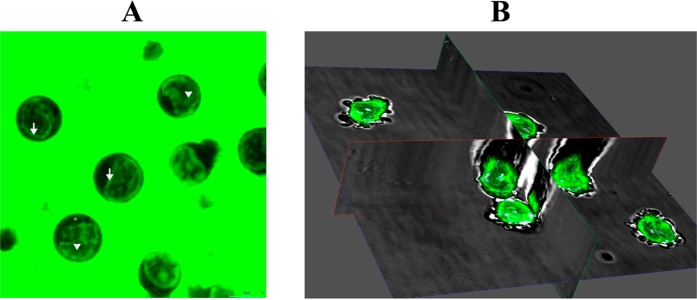
Intracellular localization of MA in peripheral blood mononuclear cells. (**A**) Optical section through the central nucleated areas of the cell. Nuclear envelope associated MA (arrows); intranuclear localization of MA (arrowheads); and diffuse MA fluorescence in the perinuclear cytoplasm (asterisk). (**B**) Three-dimensional reconstruction, from image Z-stacks, of intracellular MA distribution.

Live cell imaging showed that MA fluorescence, in the time interval studied, is seen in association with the nuclear envelope, inside the nucleus, and as a diffuse glow in the perinuclear cytoplasm (Fig. 5). It is important to mention that structural components of the cell, such as the plasma membrane or cytoskeleton, did not show pronounced accumulation of MA.

To study the initial penetration rate of MA into the cells, we performed live cell video imaging of PBMCs from the time of MA addition to the culture medium until a few seconds thereafter. To reduce the fluorescence of extracellular MA, a lower MA concentration (100 μg/ml) was used in the experiment presented. According to the data obtained, at the moment of MA’s introduction to the culture, fluorescence was completely absent. Three seconds after introduction, a weak uniformly-distributed fluorescence related to the diffusion of MA into the culture medium appeared throughout the video field, and no noticeable cellular fluorescence was observed. However, just six seconds after addition, along with an increase in the general fluorescence level in the extracellular environment, a distinctive intracellular MA distribution pattern in the cultured PBMCs was observed: accumulation of MA on the nuclear envelope; fluorescence inside the nucleus; and fluorescence of the perinuclear cytoplasm (Fig. 5).

The results indicate a high rate of initial MA penetration through the plasma membrane (within the first six seconds). Along with that, the observed intracellular localization of MA did not change significantly during the entire subsequent period of CLSM observation (up to 3 hours). Within three hours, there was also no significant increase in MA fluorescence in the identified cellular compartment.

### Evaluation of cell viability by measurement of cell membrane permeability and mitochondrial membrane potential

**Figure 6:**
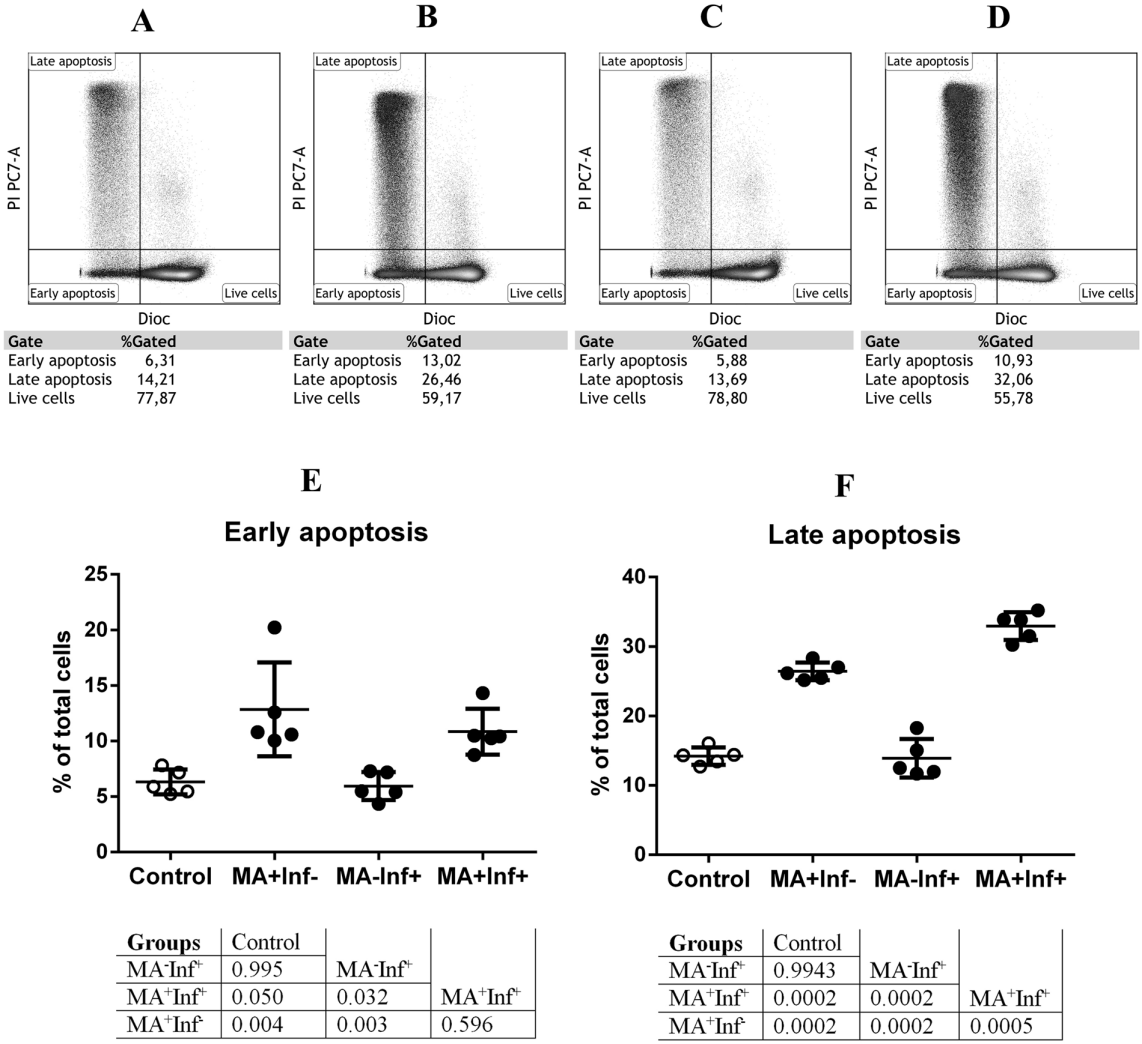
Effect of MA on apoptosis in PBMCs. Living cells were detected on the basis of a lack of PI fluorescence and a high level of DiOC6(3) fluorescence (PI-DiOC6(3)hi). Cells in the early stages of apoptosis were identified using the criterion of an absence of PI fluorescence with low DiOC6(3) fluorescence (PI-DiOC6(3)-/low). Cells in late apoptosis and/or necrosis were identified using the PI + DiOC6(3)-/low phenotype. Flow cytometry analyses of PI/ DiOC6(3) fluorescence in MA^−^Inf^−^ (**A**), MA^+^Inf^−^(**B**), MA^−^Inf^+^ (**C**), and MA^+^Inf^+^ (**D**). The DiOC6(3)/PI staining assay was used to quantify the number of apoptotic cells in early apoptosis (**E**) and late apoptosis (**F**). In the table below the graphs, the *p*-value significance levels, obtained by comparing the groups using a single-factor analysis of variance (ANOVA), followed by a pairwise comparison using the Tukey test, are indicated. Differences were considered significant when p-value < 0.05. Data are represented as mean ± SD.

Because next-generation sequencing (NGS) data indicated that MA is likely to induce apoptosis in PBMCs, we decided to measure the mitochondrial transmembrane potential (Δψm) of MA stimulated cells, both in the presence and absence of influenza infection. Reduction in ΔΨm signifies an early stage of apoptosis preceding DNA fragmentation, ROS production, and increase of membrane permeability^39^. In order to measure ΔΨm in MA^+^Inf^−^, MA^+^Inf^+^, and MA^−^Inf^+^ cells, as well as in control cells (MA^−^Inf^−^), a sensitive mitochondria-specific probe, DiOC6(3), was used^40^. Reduced ΔΨm is not an absolute indicator of apoptosis, therefore we also measured terminal apoptosis by propidium iodide staining (PI)^41,42^. Processed flow cytometry data are presented graphically in Fig. 6E,F. Raw data, including histograms of DiOC6 (3) fluorescence intensity and PI double-stain intensity, are shown in Fig. 6A–D.

Measurement of cells’ ΔΨm showed that treatment with MA led to changes in the ratios of living cells to cells in different stages of apoptosis (Fig. 6E). In particular, the fractions of cells detected in early apoptosis were: 12.9% of MA^+^Inf^−^ cells; 10.9% of MA^+^Inf^+^ cells; 5.94% of infected MA^−^Inf^+^ cells; and 6.3% of control cells (MA^−^Inf^−^). A similar pattern was also observed when measuring the fluorescence intensity of PI (Figs. 6F and S1). Under MA stimulation, the fraction of cells in late apoptosis was increased in comparison to control. In MA^+^Inf^−^ cells, the increase was by 11.8% (*p*-value = 0.0002), and in MA^+^Inf^+^ cells, the increase was by 18.7% (*p*-value = 0.0002). It was also seen that MA treatment resulted in a reduction in the number of live cells. Normal cultures (MA^−^) featured 77–79% live cells. In MA^+^Inf^−^ cells, however, that number dropped to 59.2%, and in MA^+^Inf^+^ cells, the number dropped to 55.8%.

It is worth noting that in the case of infection, MA had a greater pro-apoptotic effect, when measuring terminal apoptosis, compared to non-infected cells (MA^+^Inf^−^) (*p*-value = 0.0005). Statistically significant differences between MA^+^Inf^+^ and MA^+^Inf^−^ cells, in terms of populations in early of apoptosis, were not observed. The data obtained by flow cytometry unambiguously correlate with the results obtained by transcriptome profiling.

### Modeling of PPARα/MA and RORα/MA interactions

In order to gain atomistic insights into potential interactions between MA and PPARα, or between MA and RORα, we applied in silico approaches. First, we performed docking calculations for MA (both anionic CMA and cationic N-methylglucamine components) in the binding sites of PPARα and RORα ligand binding domains (corresponding to the sequences 202–468 and 271–523 from the UNI PROT database, respectively) in order to analyze potential interactions of both proteins with MA, as they were suggested to occur according to our experimental data and hypothesis. The binding sites for local docking were defined based on the available experimental structures of PPARα and RORα ligand binding domains in complexes with molecules known to modulate their biological activities (2-[(4-Aminomethyl)phenoxy]-2-methylpropionic acid and cholesterol, respectively). The size of clusters ranged up to 100 for AD3 and 10 for DOCK.

For CMA, we obtained 6D/3A and 2D/3A representative docking poses for PPARα and RORα, respectively (N_DOCK_/N_AD4_). For N-methylglucamine, we obtained 2_D_/2_A_ and 3_D_/3_A_ docking poses for PPARα and RORα, respectively. Then, we applied MD simulations, followed by MM-GBSA free energy calculations, in order to characterize all these obtained multiple docking poses in terms of their dynamics and energetics. In all performed simulations, proteins remained stable, which is reflected by analysis of the radius of gyration (Supplementary Table S3, Fig. S3). Apart from 2 MD simulations, wherein ligand dissociation from the binding site was observed, all protein-ligand complexes predicted by docking remained stable in terms of the distance between protein and ligand centers of mass in the course of the MD simulation (Supplementary Table S3). Standard deviations of this distance suggest that, in general, binding at the PPARα binding site is more stable than at the RORα binding site, while for RORα, CMA binding is more stable than N-methylglucamine binding.

The obtained free energy of binding values were compared to the ones from MD simulations carried out on X-ray structures of PPARα complexes and RORα complexes (Supplementary Table S4). For all simulated docking solutions for both proteins, CMA binding was weaker than the binding of ligands for which experimental structures were available. This is to be expected when taking into account an essentially smaller size of CMA in comparison to both ligands from the X-ray structures. At the same time, the obtained values for the MM-GBSA total free energy of binding are within the range of values obtained by using the same protocols for a dataset of protein complexes with glycosaminoglycans (linear anionic carbohydrates) performed on the experimental structures^43^. This suggests that, for both proteins, binding to CMA could potentially occur *in vitro* and *in vivo*. The calculated free energy values should not, however, be interpreted as absolute values, but they are useful for comparing the binding of different ligands^44^. Free energy decomposition analysis shows that, for both proteins, CMA binding is driven by hydrophobic interactions. This is similar to what is seen in the cases of ligands from the experimental structures (Supplementary Table S4). Meanwhile, in the most probable binding poses (corresponding to the lowest total energy values), electrostatic contributions are also substantial. These results suggest that, for both PPARα and RORα, CMA’s hydrophobic (acridone) and charged (acetate) moieties are both crucial for potential binding, yet with a higher importance of electrostatics in the case of PPARα.

For N-methylglucamine, the obtained MM-GBSA free energies of binding are significantly less favourable than for CMA with both PPARα and RORα (Supplementary Table S4). This suggests that CMA is more prone to be bound within the experimentally known binding pockets of PPARα and RORα than N-methylglucamine. Free energy decomposition shows that N-methylglucamine binding has significantly more favourable in vacuo electrostatic contribution to the free energy than CMA which, however, is compensated by the solvation term. The contribution of the van der Waals component to the free energy of binding is substantially less favourable for N-methylglucamine than for CMA.

In order to find out how strongly these two components of MA could be bound via ionic interactions to each other in solution, we performed an MD simulation of these two molecules in the absence of proteins. Analysis of the distance between the nitrogen atom in the amine group and the carbon atom in the carboxyl group showed that the ionic pair was formed (5 Å distance cut-off) only in 5.6% of the simulation. This finding justifies the use of the above-described docking approach in which we docked the cation and anion separately. At the same time, another scenario cannot be ruled out: first, CMA binding to the binding pockets in the protein targets and then N-methylglucamine binding on top of the binding pockets. To analyze how probable this scenario is, and because CMA was predicted to be bound more strongly than N-methylglucamine for both proteins, we performed additional docking of N-methylglucamine to both proteins (their ligand binding domains) using DOCK software. In this analysis, the receptors consisted of the proteins themselves already pre-bound to CMA. This allowed us to analyze the binding in case both parts of MA could be simultaneously bound to their potential protein interaction partners. We found that potential binding of both CMA and N-methylglucamine at the same time slightly stabilized the complexes for both proteins (Supplementary Table S4). In the case of RORα, both components of MA remain in the binding pocket.

**Figure 7:**
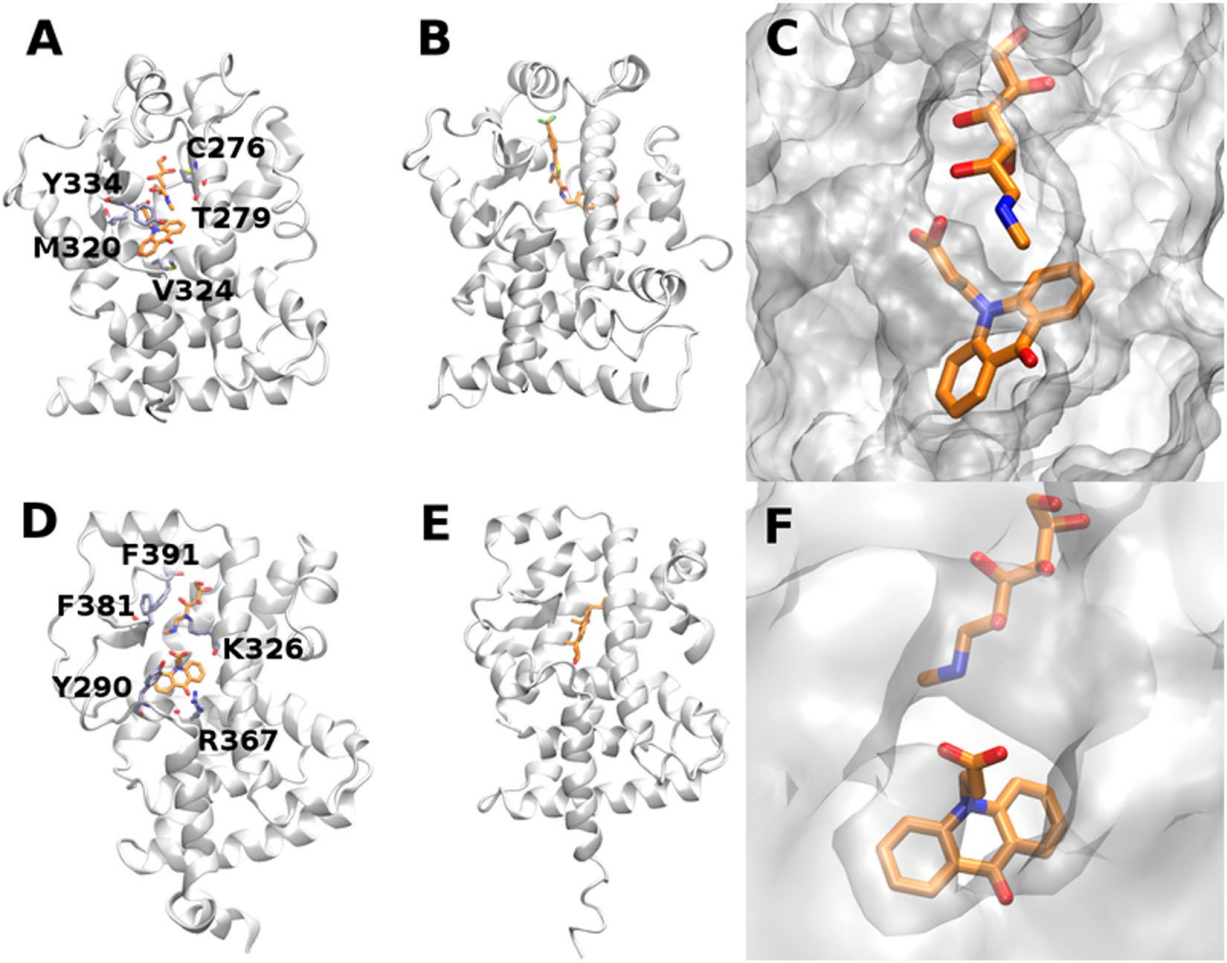
Predicted binding poses for MA/PPARα (**A–C**) and MA/RORα (**D,E**). Proteins are shown in cartoon representation. Ligands and the residues most contributing to their binding, according to per residue free energy decomposition, are shown by licorice representation (**A,D**). Surface representation of the protein active site around the bound ligand in licorice representation (**C,E**). For comparison, X-ray structures with established inhibitors are shown: PDB ID 2P54 (**B**) and 1N83 (**E**).

Furthermore, we also calculated the free energy contributions of protein residues in the systems mentioned above (Supplementary Tables S5–S7). The data show that there is a significantly higher overlap between the residues predicted to participate in CMA binding than in N-methylglucamine when compared to the corresponding data from the experimental structures. The calculated structures, featuring bound CMA and N-methylglucamine corresponding to the lowest binding energies, are shown in Fig. 7, while the behaviour of these complexes in terms of RMSD is presented in Supplementary Fig. S2. Although convergence of the RMSD was obtained, it is important to underscore that even for these most stable complexes, the ligand changed its conformation in comparison to the initial docking pose (Supplementary Fig. S4). This suggests that MD post-processing analysis of molecular docking results is necessary for these types of molecular modeling procedures. In addition, we performed H-bonding analysis for the MD simulations corresponding to the lowest binding energies for CMA and N-methylglucamine (Table 3). Several substantially stable H-bonds were observed in all these systems. Although the highest number of H-bonds was detected in the predicted RORα-N-methylglucamine complex, six of these eight H-bonds are established with the backbone of the protein and, in particular, with the oxygen atom from the carbonyl group. Similarly, for RORα-CMA, three out of four stable H-bonds are established with the protein backbone through the amide group. In case of PPARα complexes, there are more H-bonds established by the protein side chains. This might indicate a possibility that the interactions of MA within PPARα binding site could be potentially more specific than for RORα. In these terms, experimental analysis of the proteins mutated in the binding sites could be interesting for verification of the proposed model and for further understanding of the potential molecular mechanisms underlying the MA activity.

**Table 3:** H-bond analysis of the MD simulations corresponding to the lowest binding energies for CMA and N-methylglucamine (abbreviated as NMG) in complexes with PPARα and/or RORα receptors.

Protein	Ligand	Program-Pose number	H-bond description	Occupancy, %
PPARα	CMA	CMA-Autodock4.2-3	O_C=O_-Ser280(OH)	95
			O_COO_-Ala333(NH)	60
			O_COO_-Tyr334(NH)	22
	NMG	NMG-DOCK-1	O_3_-His440(NE2)	16
			H_OH4_-Gln277(OE1)	83
			N_NH_-Cys276(O_C=O_)	26
			H_OH2_-Tyr464(NE2)	13
			N_NH_-Ser280(OH)	12
RORα	CMA	CMA-DOCK-1	O_COO_-Gln289(NH)	96
			O_C=O_-Arg370(NH)	68
			O_COO_-Tyr290(NH)	60
			O_COO_-Arg370(NH2)	46
	NGM	NMG-Autodock4.2-3	N_NH_-Glu329(COO)	95
			N_NH_-Asp382(COO)	106
			H_OH4_-Tyr380(O_C=O_)	49
			H_OH_-Lys326(O_C=O_)	40
			H_OH_-Glu329(O_C=O_)	28
			H_OH1_-Lys326(O_C=O_)	24
			H_OH3_-Tyr380(O_C=O_)	20
			H_OH3_-Glu329(O_C=O_)	20

H-bonds with occupancies higher than 10% in the last 10 ns of the MD simulation are reported. In cases wherein an analyzed chemical group had identical atoms (like oxygen or hydrogen atoms in -COO or -NH2, respectively), the sum of the H-bond occupancies (corresponding to all types of H-bond) is provided, which can lead to a number higher than 100%. The number higher than 100%, however, still corresponds to a single type of the hydrogen bond in terms of the atoms participating in its establishment. For example, 106% of the N_NH_-Asp382(COO) corresponds to a unique type of hydrogen bond established between one of the two hydrogen atoms bound to the nitrogen atom in N-methylglucamine and one of the two oxygen atoms from the carboxyl group of Asp382. Since the atoms of each of these pairs are completely equivalent and are only numbered differently in the simulation for technical reasons, providing any information where these atoms are distinguished, would be physically irrelevant. The four possible combinations making up this type of the hydrogen bond would contribute 25% of the sum for all the bonds in an MD simulation of the infinite length. For NGM: hydroxyl groups are abbreviated as OH, OH1, OH2, OH3, or OH4 from the closest to the most remote group in the carbon chain in relation to the amine-group.

## Discussion

Based on transcriptome profiling data, it can be assumed that MA’s effects occur via activation of nuclear receptors (subfamily I). Using laser confocal microscopy, we have shown that the subcellular localization of MA is at the nuclear envelope, inside the nucleus, and in the perinuclear cytoplasm diffusely. Members of nuclear receptor subfamily I are transcription factors localized strictly in the cell nucleus^45,46^. Intranuclear localization of MA is consistent with its ability to manifest biological activity through binding to nuclear receptors. It should also be noted that, after a certain number of MA molecules enter the cell, the plasma membrane apparently becomes a highly effective barrier to the free diffusion of MA. Then, even over prolonged observation, a consistently high transmembrane concentration gradient of MA remains in the cell. The high diffusion rate, association with the nuclear envelope, and saturability may indicate that the transfer of MA into the nucleus is being accomplished according to the principle of facilitated translocation.

These data allow us to speculate that transmembrane transport of MA may involve more specific cellular mechanisms than plain diffusion. We suppose that MA permeation into PBMCs might be accomplished via a facilitated diffusion mechanism, under the control of cellular regulatory systems.

Presumably, MA may be an exogenous agonist of PPARα and/or RORα receptors. The natural ligands for PPARα are fatty acids and prostaglandins; for RORα, they are cholesterol and all-trans-retinoic acid (ATRA). Along with high-affinity receptors, low-affinity ligands which must be present in high concentrations for the realization of a physiological response are characteristic of nuclear receptors^47,48^. Nuclear receptors having more than one agonist and one antagonist ligand^49^ exist and are involved in a wide range of key physiological functions^50–52^. They are potential drug targets for numerous diseases and constitute an important class of therapeutic targets.

Molecular docking was performed to elucidate the mechanisms of possible binding of MA to the PPARα and RORα nuclear receptors (their ligand binding domains). Our modeling studies suggest that the CMA anion is more prone to bind to both PPARα and RORα than the N-methylglucamine cation. The calculated affinities are very similar for both proteins. The calculated free energies of binding are less favourable than for the experimental ligands used as controls but, nevertheless, the obtained values indicate that such binding could occur. CMA could potentially bind to the binding pockets of PPARα and RORα first, with N-methylglucamine subsequently binding, although its energy contribution is low. The model we calculate supports the experimental hypothesis that MA can bind to PPARα and RORα. We propose that this binding is driven by hydrophobic interactions with CMA. The residues which, according to our free energy calculations, likely participate in the modeled binding poses have been identified and their features provide potentially useful information needed to establish the molecular basis of interactions between MA and its putative protein targets.

The activation of carnitine palmitoyltransferase I (CPT1) also points to a possible interaction of MA with the nuclear PPARα receptor. CPT1 catalyzes the formation of fatty acyl carnitine for translocation across the inner mitochondrial membrane. This enzyme is strongly induced by PPARα ligands^12,53,54^.

Despite its activation of type I interferons, which we and other authors have noted^8,55^, MA actually may play an immunosuppressive role. Our results demonstrate that, when MA is processed in a heterogeneous PBMC population, cytokine signaling pathways associated with both Th1 (IL1B, IFNG) and Th2 cells (IL10, IL4/IL13) are suppressed. The increased expression of CR1, which interacts with RUNX1 and FOXP3 (Reactome ID: 8877330; genes which control the development of regulatory T lymphocytes) allows us to propose that regulatory T cells (Tregs) are activated in response to MA. Tregs are CD25^+^CD4^+^FOXP3^+^ T lymphocytes actively involved in the negative control of various aspects of the immune response^56–58^. Tregs have the ability to suppress the proliferation and function of other immune and non-immune cells including T effector cells, B cells, macrophages, dendritic cells (DCs), and osteoblasts^59^.

FOXP3 is expressed on the surface of Tregs and is a key regulator of their development and activity. It is known that binding of FOXP3 to the RUNX1 gene, which stimulates the transcription of IL2 and IFNγ, leads to the repression of those two (latter) genes^60,61^. According to our results using MA treated cells, IL2 and IFNγ expression levels remained almost unaltered compared to control cells. Also, in some differentially expressed gene scans, TGF-β and IL-10 (cytokines actively produced by Tregs) were not detected^62–65^. These facts can be explained by the rather long exposure of cells to MA. Transcriptome profiling was performed 24 hours after stimulation. By that time, the primary effects caused by MA had likely already been compensated for by counter-processes aimed at maintaining homeostasis.

Modern scientific work directly indicates that Treg activation and differentiation may be associated with activation of nuclear receptors such as: NR4A1 and NR4A2^66,67^; RORα and RORγ^59,68,69^; as well as PPARα and PPARγ^70,71^.

It is known that FOXP3 can directly bind to the AF2-activator region of the RORα and RORγt receptors via the LXXLL consensus motif^72–74^. Through its interaction with RORs, FOXP3 represses RORs’ functions and promotes Treg differentiation. The AF2 region is in the ligand binding domain of RORs and its function is directly dependent on the presence of the bound ligand^75^. Perhaps by acting as a ROR ligand, MA is involved in Treg differentiation.

In work published by the Lei group, it was demonstrated that PPARα and PPARγ agonists, in the presence of TGF-β, were able to convert human naïve CD4^+^CD25^-^ T cells into functional Tregs^71^. It has also been reported that treatment of cytokine-activated human macrophages with PPAR agonists induced apoptosis^54,76,77^.

According to the results of transcriptome profiling, MA activates the expression of genes which include the main regulators of the mitochondrial apoptosis pathway. By using flow cytometry to process MA treated cells, we have also shown that the fraction of cells showing apoptosis-related changes increases. The mechanisms of MA’s pro-apoptotic effect on PBMCs are due to a decrease in the mitochondrial transmembrane potential in the context of an increased expression of pro-apoptotic proteins. In these lines, the initiation of apoptosis occurs even without activation of the TCR as happens, for example, during influenza infection. However, in late stage apoptosis, MA had the strongest apoptogenic effect on cells infected with influenza virus.

## Materials and Methods

### PBMC preparation

Peripheral blood mononuclear cells (PBMCs) were isolated from healthy donor buffy coat blood samples (50 ml) using ficoll density gradient centrifugation, Lymphosep medium (Biowest), and Leucosep tubes (Greiner Bio-One). After separation, PBMCs were frozen in aliquots using a freezing container (Nalgene) and stored in liquid nitrogen. For experiments, PBMCs were suspended in DMEM culture media (Gibco, USA) containing 5% FBS (Gibco, USA) and transferred to 24-well plates (9 × 10^6^ cells/well). Cells were incubated overnight (37 °C, 5% CO_2_), washed twice with DMEM media, and were subsequently infected with influenza virus at a multiplicity of infection (MOI) of 0.001. The H1N1 pdm09 human influenza isolate A/California/07/09 was obtained from Smorodintsev Research Institute of Influenza collection. After 1 hour of virus adsorption, inoculum was removed, and cells were washed twice with DMEM media. After washing, cells were allowed to grow in DMEM containing 2.5 mg/ml MA for 24 hours.

All study and its protocols were approved by the local institutional Ethics Committee (protocol № 113, dated March 03, 2017). All experiments with PBMCs were performed in accordance with the relevant guidelines and regulations.

### RNA extraction

Total RNA was isolated from 100–200 μl cell suspensions (10^5^–10^6^) using the RNeasy Mini Kit (Qiagen ^#^74106, USA) with full adherence to the manufacturer’s recommendations. After extraction, RNA concentrations were measured with a NanoDrop ND-1000 spectrophotometer (Thermo Fisher Scientific, USA); in order to evaluate the purity of isolates, A_260/280_ ratios (normal ≥1.9) were also determined. Total RNA was characterized by capillary electrophoresis using the Agilent RNA 6000 Nano Kit (^#^5067-1511) on an Agilent 2100 Bioanalyzer (Agilent, USA).

### Library preparation and transcriptome sequencing

cDNA libraries for next-generation sequencing were generated using an NEBNext Ultra Directional RNA Library Prep Kit for Illumina (NEB ^#^E7420S, USA). For cDNA library preparations, 1000 ng of total RNA (as quantified by Bioanalyzer) was used as starting material. For labeling of cDNA libraries, the dual index primer strategy was used (NEBNext i501-502 and NEBNext i701-704 primers). The quality and concentration of the obtained libraries were evaluated by capillary electrophoresis using a High Sensitivity DNA Chip kit on an Agilent 2100 Bioanalyzer (Agilent, USA) and also by measurement with a Nanodrop-2000 fluorescent reader using the PicoGreen Assay for dsDNA kit (Thermo, ^#^P7589, USA). The cDNA libraries obtained from eight RNA samples were mixed with equimolar ratios. The concentration of the pooled library was normalized by PCR using the NEBNext Library Quant Kit for Illumina (NEB, USA). In order to generate clusters, a 4 nM pooled DNA library was applied to the Illumina flow cell (MiSeq System Denature and Dilute Libraries Guide, Illumina, USA). Sequencing was performed according to Illumina paired-end read sequencing protocols on a MiSeq device (Illumina, USA) using the MiSeq Reagent Kit v3 (150-cycles).

### Read mapping and analysis of differential gene expression

FastQ sequencing datasets were trimmed and filtered in AfterQC software in automatic mode^78^. Quantification and quality control checks were performed with FastQC^79^. Paired-end reads were aligned to the human reference genome (GRCh38.p3) using STAR Aligner v2.5.1b^80^ and default settings. Gencode v23^81^ was used to assign genomic coordinates to sequence reads. Analysis of differentially expressed genes was performed with the R v3.3.1 free software environment^82^, Rstudio^83^, and the DESeq2^84^ package. Gene quantification was obtained using the FeatureCounts tool^85^. For plotting and visualization, R and Phantasus^86^ were used. The Reactome database^11^ was used to interpret and visualize biological pathways and processes that related to differentially expressed genes.

### Real-time quantitative PCR (RT-qPCR)

Two micrograms of total RNA were reverse transcribed using oligo-dT^16^ primers and MMLV reverse transcriptase (Promega, USA). Monoplex and multiplex TaqMan PCR assays developed at the Smorodintsev Research Institute of Influenza were used to verify NGS results^87^. PCR was performed with a CFX96 thermocycler (Bio-Rad, USA) with signal recording at the appropriate wavelengths. For estimation of viral RNA in samples, we used Centers for Disease Control and Prevention (CDC, USA) recommended primers for typing group A influenza viruses and human RNase P. Real-time PCR was performed using the SuperScript III Platinum One-Step qRT-PCR Kit (Invitrogen, USA) according to CDC recommendations. The primer and TaqMan probe sequences used in this work are supplied in the Supplementary Table S1. Relative expression of cytokines were calculated by the ΔΔCt method^88^ using RNase P (for influenza A) and GAPDH (for all other genes) as the genes used for normalization.

#### Microscopy

For confocal live cell imaging, samples of 10^5^ PBMCs in DMEM (Thermo Fisher Scientific, USA) supplemented with 10% Fetal Bovine Serum (Biowest, France) were placed in Nunc Lab-Tek II Chambered Coverglass slide containers (Thermo Fisher Scientific, USA). After 24 hours of incubation (37 °C, 5% CO_2_), Cycloferon (Polysan, Russia) was added to the cells at a final concentration of 500 µg/ml; incubations (cells with Cycloferon) ranged from 0 to 3 hours. Confocal microscopy of cells was performed using a TCS SP8 inverted confocal laser scanning microscope (Leica, Germany). A 405 nm diode laser was used to excite MA fluorescence. Fluorescence detection was performed using a photomultiplier tube (PMT) in the spectral range of 415–550 nm.

### Flow cytometry

In order to evaluate mitochondrial transmembrane potential (Δψm), 20x stock DiOC6(3) reagent (Invitrogen, USA) was added to 100 μl cell suspensions (2 × 10^6^ cells/ml) such that final dye concentrations were 20 nM. After addition of dye, samples were thoroughly mixed and incubated for 20 minutes (37 °C, 5% CO_2_) in darkness. Following incubation, samples were washed with excess PBS and centrifuged for 10 minutes at 500 g. Next, supernatants were removed, and cell pellets were resuspended in 100 μl of fresh PBS. Ten microliters of propidium iodide (PI) solution (Sigma-Aldrich, USA) was added to the resulting cell suspensions, giving final PI concentrations of 1 μg/ml, and samples were then incubated for 10 minutes at room temperature in darkness. Following incubation, 200 μl of PBS was added to the samples, and cytometric counts were performed. For each of the samples, at least 50,000 single cells were analyzed; data analysis was performed using Kaluza software (Beckman Coulter, USA).

### Molecular docking

The DOCK^89^ and Autodock 4.2^90^ programs were used to predict the position and conformation (binding pose) of MA ligand in the experimentally known binding pockets of PPARα and RORα. The available crystal structures of the human PPARα (PDB ID: 2P54, 1.79 Å)^91^ and RORα (PDB ID: 1N83, 1.63 Å)^92^ ligand binding domains complexes were used for obtaining the structures of the corresponding receptors for docking. The bound ligands (cholesterol and GW735) have been removed from the receptor structures prior to docking. For both programs, the grid was centered on the ligand binding site obtained from the experimentally determined ligand-protein complexes.

In Autodock 4.2, docking with a rigid receptor with Gasteiger charges and a flexible ligand with AM1-BCC partial charges obtained in the Antechamber package distributed with AMBER 16^93,94^ was performed with a grid spacing of 0.375 Å with a grid box length of 22.5 Å (60 grid points for each dimension). The Lamarckian genetic algorithm was used with: an initial population size of 150; 27 × 10^3^ generations; 2.5 × 10^6^ energy evaluations; and 100 independent runs. These default parameters are considered to be sufficient for ligands with the number of degrees of freedom less than or equal to eleven^90^.

In DOCK 6, atom types were assigned and hydrogens were added for the ligands using Chimera^95^. Subsequently, AM1-BCC partial charges were calculated using the Antechamber package from AMBER 16^93,94^. For receptor molecules, all water molecules were removed, while hydrogens were added using Chimera^95^, and AMBER partial charges were assigned. No additional optimization of the protein structures was carried out. The GRID program of DOCK was used to precalculate scoring function potential grids^96^; all parameters were set to default values. For matching, the dms program was used to generate a molecular surface for each receptor. The SPHGEN package of DOCK was used to create a negative image of the surface using spheres^97,98^. All spheres found within 10 Å of crystal ligand atoms were selected. The receptor box restricting the binding site was calculated with the SHOWBOX program using a box length of 8 Å. All docking runs described in this section involved rigid docking procedures. These default parameters have been shown to be sufficient for significantly bigger ligands, in terms of the number of degrees of freedom, than the ones used in this study^99^.

Cluster analysis was performed for 100 obtained docking poses using the DBSCAN algorithm^100^ with a neighborhood search radius of 2.5 Å and the number of cluster members exceeding 5. From each cluster, representative docking solutions corresponding to different rotameric states of the ligands with the lowest (most favourable) docking scores were chosen for further analysis.

### Molecular dynamics

Molecular dynamics (MD) simulations of the MA/PPARα and MA/RORα complexes (obtained by molecular docking calculations), as well as of PPARα and RORα ligand binding domains crystal structures with experimentally established inhibitors (PDB ID: 2P54 and 1N83), were carried out in AMBER 1694. All non-proteinic ligand molecules (CMA, N-methylglucamine, 2-[(4-Aminomethyl)phenoxy]-2-methylpropionic acid and cholesterol) were parameterized using a default procedure as implemented in the AMBER MD package^94^. In particular, the initial ligand structures were either built in the LEAP module of AMBER (CMA and N-methylglucamine) or obtained from X-ray structures (2-[(4-Aminomethyl)phenoxy]-2-methylpropionic acid and cholesterol). Then, ANTECHAMBER scripts were used to assign atomic type, covalent bond, angle and dihedral angle parameters to ligand atoms from the general AMBER force field (GAFF)^101^ which is compatible with the ff14SB force field used for the proteinic part of the analyzed system. The AM1-BCC charge model was used to assign the partial charges of these ligands^93,102^. Each complex was solvated in a truncated octahedron TIP3P water periodic box with a distance between the periodic box walls and solute atoms of 10 Å. Next, complexes were neutralized by counterions. The ff14SB and GAFF force field parameters were used for protein and non-proteinic ligand molecules, respectively. MD simulations were preceded by two energy-minimization steps: 1500 cycles of steepest descent and 1000 cycles of conjugate gradient with1 harmonic force restraints of 10 kcal/(mol·Å^2^) applied to protein and ligand atoms; then, 3000 cycles of steepest descent and 3000 cycles of conjugate gradient without restraints. This was followed by heating of the system from 0 to 300 K for 10 ps with harmonic force restraints of 10 kcal/(mol·Å^2^) applied to protein and ligand atoms, and a 100 ps MD equilibration run at 300 K and 10^5^ Pa in an NPT ensemble.

Following the equilibration procedure, 20 ns MD production runs were performed under periodic boundary conditions in an NPT ensemble. An 8 Å cut-off was applied to treat non-bonded interactions, and the Particle Mesh Ewald method was applied to treat long-range electrostatic interactions. Analysis of the MD simulations was performed using the CPPTRAJ module of AMBER. Energetic post-processing of the trajectories and per residue energy decomposition were performed for protein-organic molecule complexes using Molecular Mechanics-Generalized Born Surface Area (MM-GBSA) with the model igb = 2. This analysis was carried out for the following parts of the trajectories: (i) the last 10 ns of the simulation if convergence in terms of ligand RMSD was reached; or (*ii*) the last part of the simulation when the RMSD of the ligand converged; or (*iii*) for the frames when the protein-ligand complex was stable before dissociation from the binding site was observed. Visual analysis of the trajectories was carried out using VMD^103^. For H-bond analysis, protein-ligand H-bond averaged parameters were calculated, using CPPTRAJ default parameters (the distance between heavy atoms less than 3.0 Å, angle greater than 135°), for the last 10 ns of the MD simulation. Radius of gyration and RMSD in the MD simulation were analyzed using default approaches implemented in CPPTRAJ. In order to characterize the strength of ionic interactions between the N-methylglucamine cation and the CMA anion, we carried out a 100 ns MD simulation in which those two molecules were unrestrained in a truncated octahedron TIP3P water periodic box with a distance between the periodic box walls and the solute atoms of 15 Å starting from the conformation corresponding to the presence of the salt bridge.

### Ethics

The study and its protocols were approved by the Smorodintsev Research Institute of Influenza institutional Ethics Committee (protocol № 113, dated March 03, 2017). Serum specimens used for isolation of peripheral blood mononuclear cells were provided by the blood transfusion station (Contract Number 128_15092017). Informed consent was obtained from all donors who provided research materials. All biological experiments were performed in accordance with the relevant guidelines and regulations.

## Supplementary information

Supplementary information
