# Supplementary material for: Meglumine acridone acetate, the ionic salt of CMA and N-methylglucamine, induces apoptosis in human PBMCs via the mitochondrial pathway
Source: Sci Rep. 2019 Dec 3;9:18240. doi: 10.1038/s41598-019-54208-9 (PMC6890692; doi:10.1038/s41598-019-54208-9)

**Supplementary information**

1. **Meglumine acridone acetate, the ionic salt of CMA and *N*-methylglucamine, induces apoptosis in human PBMCs via the mitochondrial pathway**
2. Marina A. Plotnikova^1,*^, Sergey A. Klotchenko^1^, Artem A. Kiselev^2^, Andrey N. Gorshkov^1^, Anna-Polina S. Shurygina^1^, Kirill A. Vasilyev^1^, Urszula Uciechowska-Kaczmarzyk^3^, Sergey A. Samsonov^3^, Alexey L. Kovalenko^4^, Andrey V. Vasin^1^
3. 1: Smorodintsev Research Institute of Influenza, St. Petersburg, Russia
4. 2: Almazov National Medical Research Centre, St. Petersburg, Russia
5. 3: Faculty of Chemistry, University of Gdańsk, Gdańsk, Poland
6. 4: Institute of Toxicology, Federal Medical-Biological Agency of Russia, St. Petersburg, Russia
7. * Corresponding author: [marina.plotnikova@influenza.spb.ru](mailto:marina.plotnikova@influenza.spb.ru); [biomalinka@gmail.com](mailto:biomalinka@gmail.com)

# Supplementary information

**Table S1.** Primers used for real-time quantitative PCR analysis.

| **Gene** | **Type** | **Sequence (5'–>3')** | **Tm, °C** |
| --- | --- | --- | --- |
| **IL24** | Forward primer | GTGATGAGGAGCTGCTTTCG | 58.99 |
|  | Reverse primer | ATAGCAGAAACCGCCTGTGT | 59.68 |
|  | Internal oligo | ***FAM-***CGGCTGCTGCAGCAGGAGGT***-BHQ1*** | 61.77 |
| **RNASE1** | Forward primer | TCTCTCAGACACCAAGCTGC | 59.68 |
|  | Reverse primer | TCATATTCCGGCGCCTCATC | 60.04 |
|  | Internal oligo | ***FAM-***TTCCCCCAGCAGCAGCTCCA***-BHQ1*** | 60.11 |
| **RNASE6** | Forward primer | CTTCCCCTTCCCTATACACACA | 58.89 |
|  | Reverse primer | AGCCTTGGTGAGACGCTTAG | 59.75 |
|  | Internal oligo | ***FAM-***TCAGCAGGAGCCCCAACACTGA***-BHQ1*** | 59.79 |
| **IFN-γ** | Forward primer | GCTTTTCAGCTCTGCATCGT | 59.20 |
|  | Reverse primer | CCGCTACATCTGAATGACCTG | 58.52 |
|  | Internal oligo | ***CY5-***TGGCTGTTACTGCCAGGACCCA***-BHQ2*** | 59.78 |
| **IL-1β** | Forward primer | AGCTGATGGCCCTAAACAGA | 58.71 |
|  | Reverse primer | TGGTGGTCGGAGATTCGTAG | 58.90 |
|  | Internal oligo | ***HEX-***GCCCTCTGGATGGCGGCATC***-BHQ1*** | 59.90 |
| **IL-2** | Forward primer | AAAGAAAACACAGCTACAACTGG | 57.75 |
|  | Reverse primer | GAAGATGTTTCAGTTCTGTGGC | 57.85 |
|  | Internal oligo | ***CY5-***TGTGAGCATCCTGGTGAGTTTGGG-***BHQ2*** | 64.91 |
| **IL-18** | Forward primer | AAACTATTTGTCGCAGGAATAAAGAT | 57.72 |
|  | Reverse primer | GCTTGCCAAAGTAATCTGATTCC | 58.32 |
|  | Internal oligo | ***ROX-***TGCAATTGTCTTCTACTGGTTCAGCAGC***-BHQ2*** | 65.80 |
| **IL-10** | Forward primer | TCCCTGTGAAAACAAGAGCAAG | 59.05 |
|  | Reverse primer | CTCATGGCTTTGTAGATGCCT | 58.07 |
|  | Internal oligo | ***HEX-***CCGTGGAGCAGGTGAAGAATGCC***-BHQ1*** | 60.06 |
| **NFkB** | Forward primer | gctcagtgagcccatggaat | 60.11 |
|  | Reverse primer | tgatgctcttgaaggtctcatatgtc | 60.68 |
|  | Internal oligo | ***FAM-***tcaccggattgaggagaaac***-BHQ-1*** | 57.24 |
| **InfA** | Forward primer | GACCRATCCTGTCACCTCTGAC | 60.00 |
|  | Reverse primer | AGGGCATTYTGGACAAAKCGTCTA | 60.00 |
|  | Internal oligo | ***FAM-***TGCAGTCCTCGCTCACTGGGCACG***-BHQ1*** | 60.00 |
| *Endogenous controls* | | | |
| **RNase P** | Forward primer | AGATTTGGACCTGCGAGCG | 60.45 |
|  | Reverse primer | GAGCGCCTGTCTCCACAAGT | 62.44 |
|  | Internal oligo | ***FAM-***TTCTGACCTGAAGGCTCTGCGCG***-BHQ1*** | 67.21 |
| **GAPDH** | Forward primer | CAGTCAGCCGCATCTTCTTTTGCGTCG | 68.59 |
|  | Reverse primer | CAGAGTTAAAAGCAGCCCTGGTGACCAGG | 68.84 |
|  | Internal oligo | ***FAM-***TGGGGAAGGTGAAGGTCGGAGTCAACGGATTTGGTC***-BHQ1*** | 74.15 |

**Table S2.** Distribution of tags from libraries.

|  | MA^-^Inf^−^ | | MA^+^Inf^−^ | | MA^-^Inf^+^ | | MA^+^Inf^+^ | |
| --- | --- | --- | --- | --- | --- | --- | --- | --- |
|  | Rep1 | Rep2 | Rep1 | Rep2 | Rep1 | Rep2 | Rep1 | Rep2 |
| Total Reads (single-end) | 2258459 | 2234820 | 2309644 | 3188508 | 2899385 | 3578312 | 3412273 | 4841246 |
| GC Content (%) | 48 | 47 | 48 | 48 | 48 | 48 | 48 | 48 |
| %≥Q30 (%) | 97.3 | 97.1 | 97.3 | 97.4 | 97.3 | 97.3 | 97.4 | 97.4 |
| Mapped Ratio (%) | 88.9 | 89.5 | 89 | 89.2 | 90.3 | 90.8 | 89.4 | 89.8 |
| Unique Mapped Ratio (%) | 76.7 | 77.8 | 76.6 | 77.3 | 79.3 | 97.4 | 77.5 | 77.6 |

**Table S3.** Stability of protein-ligand complexes by MD simulation.

| Protein | Ligand-Program-Pose number/X-ray | ^*^R gyration (protein), Å | ^**^Distance (CM_prot_-CM_lig_), Å |
| --- | --- | --- | --- |
| **PPARα** | CMA-Autodock4.2-1 | 19.3±0.1 | 9.0±0.3 |
|  | CMA-Autodock4.2-2 | 19.4±0.1 | 8.3±0.4 |
|  | CMA-Autodock4.2-3 | 19.4±0.1 | 7.8±0.3 |
|  | CMA-Autodock4.2-4 | 19.4±0.1 | 8.5±0.4 |
|  | CMA-Autodock4.2-5 | 19.3±0.1 | 6.7±0.5 |
|  | CMA-Autodock4.2-6 | 19.6±0.1 | 9.1±0.4 |
|  | CMA-DOCK-1 | 19.4±0.1 | 14.0±0.5 |
|  | CMA-DOCK-2 | 19.3±0.1 | 9.0±0.3 |
|  | NMG-Autodock4.2-1 | 19.4±0.2 | ^***^11.4±2.8 |
|  | NMG-Autodock4.2-2 | 19.3±0.2 | 9.1±0.4 |
|  | NMG-DOCK-1 | 19.5±0.1 | 9.0±0.6 |
|  | NMG-DOCK-2 | 19.3±0.2 | 6.3±0.3 |
|  | CMA+NMG-DOCK | 19.4±0.1 | 7.6±0.3 |
|  | X-ray (2P54) | 19.3±0.1 | 10.5±0.3 |
| **ROR**α | CMA-Autodock4.2-1 | 19.8±0.3 | 9.9±0.4 |
|  | CMA-Autodock4.2-2 | 19.7±0.3 | 9.0±0.3 |
|  | CMA-Autodock4.2-3 | 19.8±0.2 | 12.9±0.6 |
|  | CMA-DOCK-1 | 19.9±0.2 | 8.7±0.3 |
|  | CMA-DOCK-2 | 19.9±0.1 | 10.7±0.8 |
|  | CMA-DOCK-3 | 19.6±0.2 | 9.7±0.8 |
|  | NMG-Autodock4.2-1 | 19.7±0.2 | 11.1±0.6 |
|  | NMG-Autodock4.2-2 | 19.5±0.2 | ^***^16.2±2.1 |
|  | NMG-Autodock4.2-3 | 19.7±0.3 | 9.8±0.6 |
|  | NMG-DOCK-1 | 19.8±0.2 | 11.9±1.1 |
|  | NMG-DOCK-2 | 19.8±0.3 | 9.3±1.1 |
|  | NMG-DOCK-2 | 19.8±0.2 | 10.2±1.2 |
|  | CMA+NMG-DOCK | 19.4±0.1 | 11.8±0.9 |
|  | X-ray (1N83) | 19.6±0.2 | 10.2±0.4 |

^*^Radius of gyration is calculated for the whole MD simulation.

^**^Distance between the protein and ligand centers of mass is calculated for the last 10 ns of the MD simulation in order to account for the equilibrated binding pose and its standard deviation. In case of two ligands (CMA+NMG), the center of mass of ligand was defined as a common center of mass of both molecules.

^***^The ligand dissociated from the binding site in the course of MD simulation.

**Table S4.** MM-GBSA free energy analysis of the predicted complexes of PPARα and RORα with CMA and N-methylglucamine (abbreviated as NMG), in comparison to their complexes with ligands for which experimental structures are available.

| Protein | Ligand-Program-Pose number/X-ray | ΔG_total_, kcal/mol | ΔG_vdw_, kcal/mol | ΔG_Ele_, kcal/mol |
| --- | --- | --- | --- | --- |
| **PPAR**α | CMA-Autodock4.2-1 | -24.9±2.6 | -32.7±1.9 | 1.9±11.3 |
|  | CMA-Autodock4.2-2 | -22.3±2.6 | -31.8±2.3 | -2.4±10.4 |
|  | CMA-Autodock4.2-3 | -26.5±2.8 | -33.3±3.0 | -23.8±12.1 |
|  | CMA-Autodock4.2-4 | -22.3±2.6 | -32.3±2.2 | -19.9±14.2 |
|  | CMA-Autodock4.2-5 | -19.4±2.5 | -31.5±3.0 | -8.6±8.8 |
|  | CMA-Autodock4.2-6 | -16.8±3.2 | -30.3±2.2 | -0.5±11.1 |
|  | CMA-DOCK-1 | -20.0±2.4 | -32.3±2.0 | 9.9±8.6 |
|  | CMA-DOCK-2 | -24.3±3.6 | -37.9±2.2 | 4.8±8.8 |
|  | NMG-Autodock4.2-1 | -1.4±1.9 | -10±3.3 | -69.7±13.0 |
|  | NMG-Autodock4.2-2 | -13.7±2.7 | -22.6±2.4 | -44.4±8.6 |
|  | NMG-DOCK-1 | -17.5±2.1 | -22.8±2.3 | -64±8.6 |
|  | NMG-DOCK-2 | -14.5±2.5 | -25±2.1 | -31.4±7.7 |
|  | CMA+NMG-DOCK | -35.0±3.1 | -54.6±2.5 | -39.8±6.1 |
|  | X-ray (2p54) | -52.1±3.0 | -57.8±2.9 | -32.2±5.8 |
| **ROR**α | CMA-Autodock4.2-1 | -14.1±2.0 | -30.5±1.8 | 58.4±6.3 |
|  | CMA-Autodock4.2-2 | -15.1±1.9 | -31.6±2.0 | 55.5±5.8 |
|  | CMA-Autodock4.2-3 | -16.8±1.7 | -34.5±1.8 | 26.4±6.7 |
|  | CMA-DOCK-1 | -28.1±2.4 | -30.0±3.0 | -7.1±15.1 |
|  | CMA-DOCK-2 | -21.5±2.2 | -28.9±2.3 | 49.9±10.4 |
|  | CMA-DOCK-3 | -26.7±3.3 | -30.4±2.0 | 17.7±15.4 |
|  | NMG-Autodock4.2-1 | -7.8±2.4 | -22.0±1.4 | -110.5±7.3 |
|  | NMG-Autodock4.2-2 | -10.3±4.6 | -5.6±3.9 | -159.7±12.8 |
|  | NMG-Autodock4.2-3 | -22.6±4.9 | -13.4±3.6 | -230±26.7 |
|  | NMG-DOCK-1 | -7.7±2.2 | -21.6±1.7 | -77.4±7.7 |
|  | NMG-DOCK-2 | -7.4±2.1 | -20.2±2.1 | -100.2±7.3 |
|  | NMG-DOCK-2 | -12.3±2.2 | -23.4±1.8 | -97.2±7.3 |
|  | CMA+NGG-DOCK | -32.0±4.3 | -53.2±3.0 | -58.7±11.4 |
|  | X-ray (1n83) | -55.3±2.5 | -60.1±2.2 | -7.5±1.9 |

**Table S5.** MM-GBSA per residue free energy decomposition: CMA.

| Protein | Program-Pose number/X-ray | Residues most contributing to binding  (free energy impact on binding lower than -1 kcal/mol ) |
| --- | --- | --- |
| **PPAR**α | Autodock4.2-1 | L321, V332, T279, Y334, A333, V324, M330 |
|  | Autodock4.2-2 | V332, Y334, L321, A333, T279 |
|  | Autodock4.2-3 | V332, L321, A333, Y334, T279, I317, M320 |
|  | Autodock4.2-4 | V332, T279, C275, L321, M355, A333, M330, S280 |
|  | Autodock4.2-5 | L321, C275, S280, M330, T279 |
|  | Autodock4.2-6 | V332, T279, M355, C276, M330, L321 |
|  | DOCK-1 | V332, C275, T279, M330, A333, C275 |
|  | DOCK-2 | Q277, T279, C275, M355, M330, I354 |
|  | X-ray (2p54) | Q277, C275, H440, C275, V332, M355, T279, I339, M330, F273, I354, I272, Y314 |
| **ROR**α | Autodock4.2-1 | I325, M368, K324 |
|  | Autodock4.2-2 | I325, K324, C323, M368, V363 |
|  | Autodock4.2-3 | I325, M368, F365, V403, V363, K324, F381, R367 |
|  | DOCK-1 | R367, K324, M368, Y290 |
|  | DOCK-2 | R370, R367, Y333, A330, Y380, Y290 |
|  | DOCK-3 | K324, F381, M368, D382 |
|  | X-ray (1n83) | I325, M368, V379, V363, C323, A330, R367, F391, V403 |

**Table S6.** MM-GBSA per residue free energy decomposition: N-methylglucamine.

| Protein | Program-Pose number/X-ray | Residues most contributing to binding  (free energy impact on binding lower than -1 kcal/mol ) |
| --- | --- | --- |
| **PPAR**α | Autodock4.2-1 | N217, K216 |
|  | Autodock4.2-2 | Y314, S280 |
|  | DOCK-1 | S280, C276, Q277, M355 |
|  | DOCK-2 | Q277, M355, C276, I317, S280, T279 |
|  | X-ray (2p54) | Q277, C275, H440, C275, V332, M355, T279, I339, M330, F273, I354, I272, Y314 |
| **ROR**α | Autodock4.2-1 | Y380, I327, K326, F381 |
|  | Autodock4.2-2 | K326 |
|  | Autodock4.2-3 | D382, E329, A330, F381, Y380, Y333 |
|  | DOCK-1 | C396, C323, I327, |
|  | DOCK-2 | Y380, V379, V403 |
|  | DOCK-3 | F399, I327, F391 |
|  | X-ray (1n83) | I325, M368, V379, V363, C323, A330, R367, F391, V403 |

**Table S7.** MM-GBSA per residue free energy decomposition: CMA and N-methylglucamine (abbreviated as NMG).

| Protein | Program-Pose number/X-ray | Residues most contributing to binding  (free energy impact on binding lower than -1 kcal/mol ) |
| --- | --- | --- |
| **PPAR**α | CMA+NMG-DOCK | Y334, V332, A333, M320, C276, I317, L321, M330, M220, T279, V324 |
|  | X-ray (2p54) | Q277, C275, H440, C275, V332, M355, T279, I339, M330, F273, I354, I272, Y314 |
| **ROR**α | CMA+NMG-DOCK | K326, R367, M368,F381, C323, L322, F391, Y290 |
|  | X-ray (1n83) | I325, M368, V379, V363, C323, A330, R367, F391, V403 |


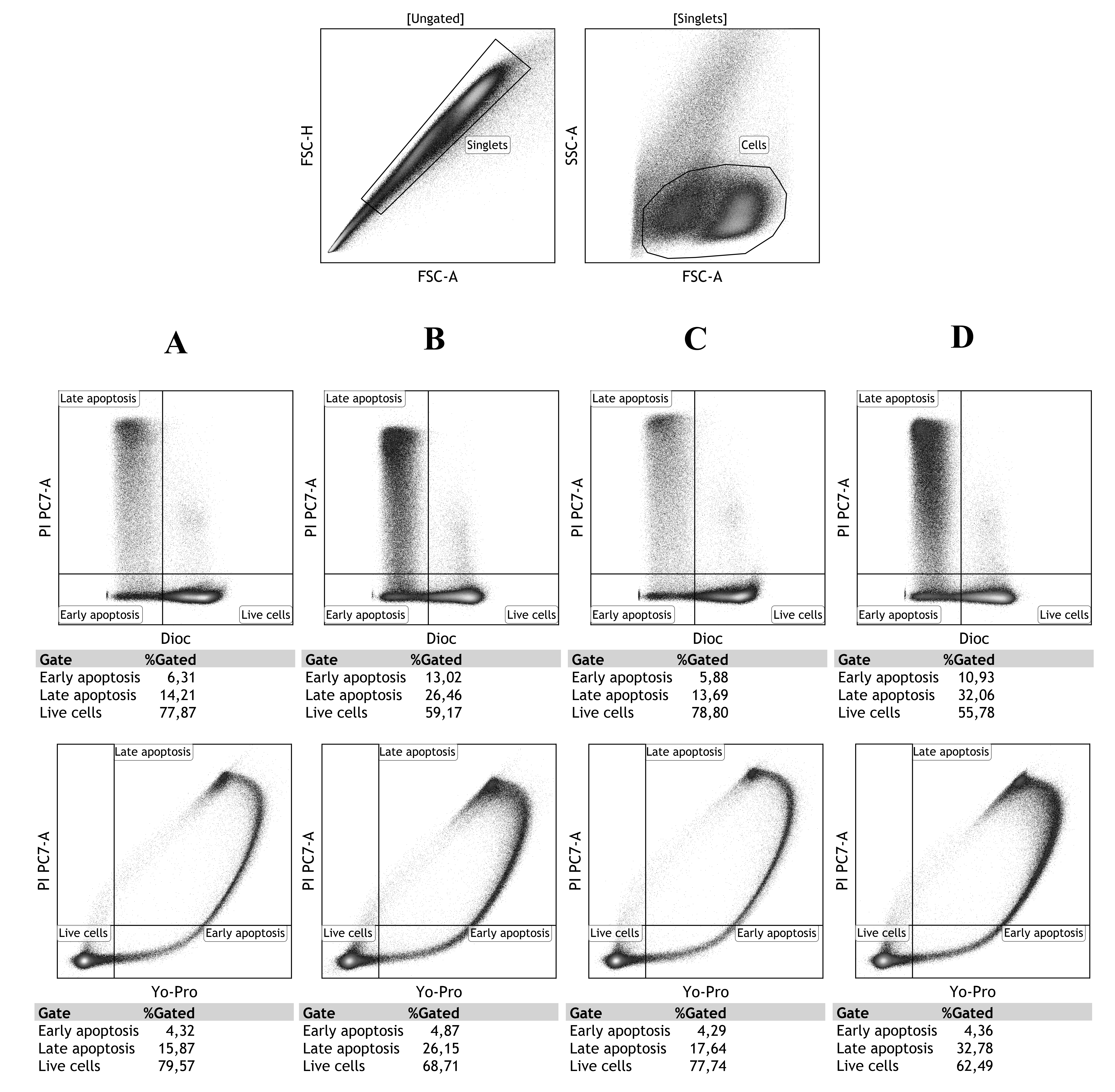
**Figure S1 Effect of MA on apoptosis in PBMCs** Flow cytometry analyses of PI/ DiOC6(3) fluorescence and YO-PRO-1and PI labeling fluorescence in MA^-^Inf^-^ (A), MA^+^Inf^-^(B), MA^-^Inf^+^ (C), and MA^+^Inf^+^ (D).

**Figure S2. All-atom RMSD for receptor (black) and ligand (red) obtained by MD simulations corresponding to the lowest binding energies for CMA and N-methylglucamine (abbreviated as NMG) in complexes with PPA**Rα **and/or RO**Rα **receptors.** Prior to RMSD calculations, the trajectory was centered on the receptor molecule. The y-axis range was chosen to maintain consistency between the graphs for all four systems. Therefore, the upper range corresponds to the maximum RMSD value from all analyzed simulations.


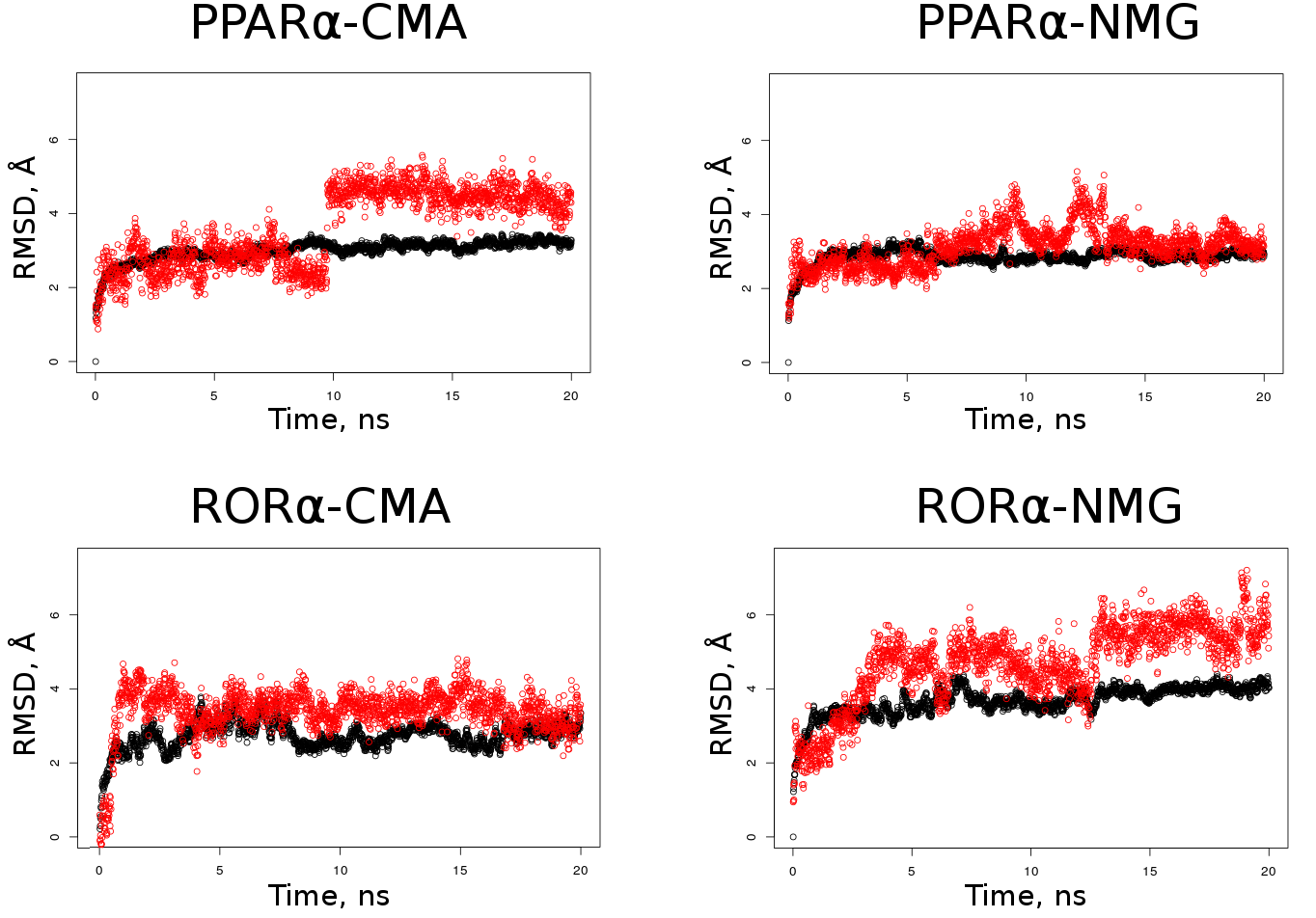


**Figure S3. Radii of gyration (left) and protein-ligand center of mass distances (right) obtained by MD simulations corresponding to the lowest binding energies for CMA and N-methylglucamine (abbreviated as NMG) in complexes with PPA**Rα **and/or RO**Rα **receptors.**  The y-axis range was chosen to maintain consistency between the graphs for all four systems. For protein-ligand distance, the data for the last 10 ns (this part of the trajectory was used for the free energy calculations) of the trajectory are provided.

**
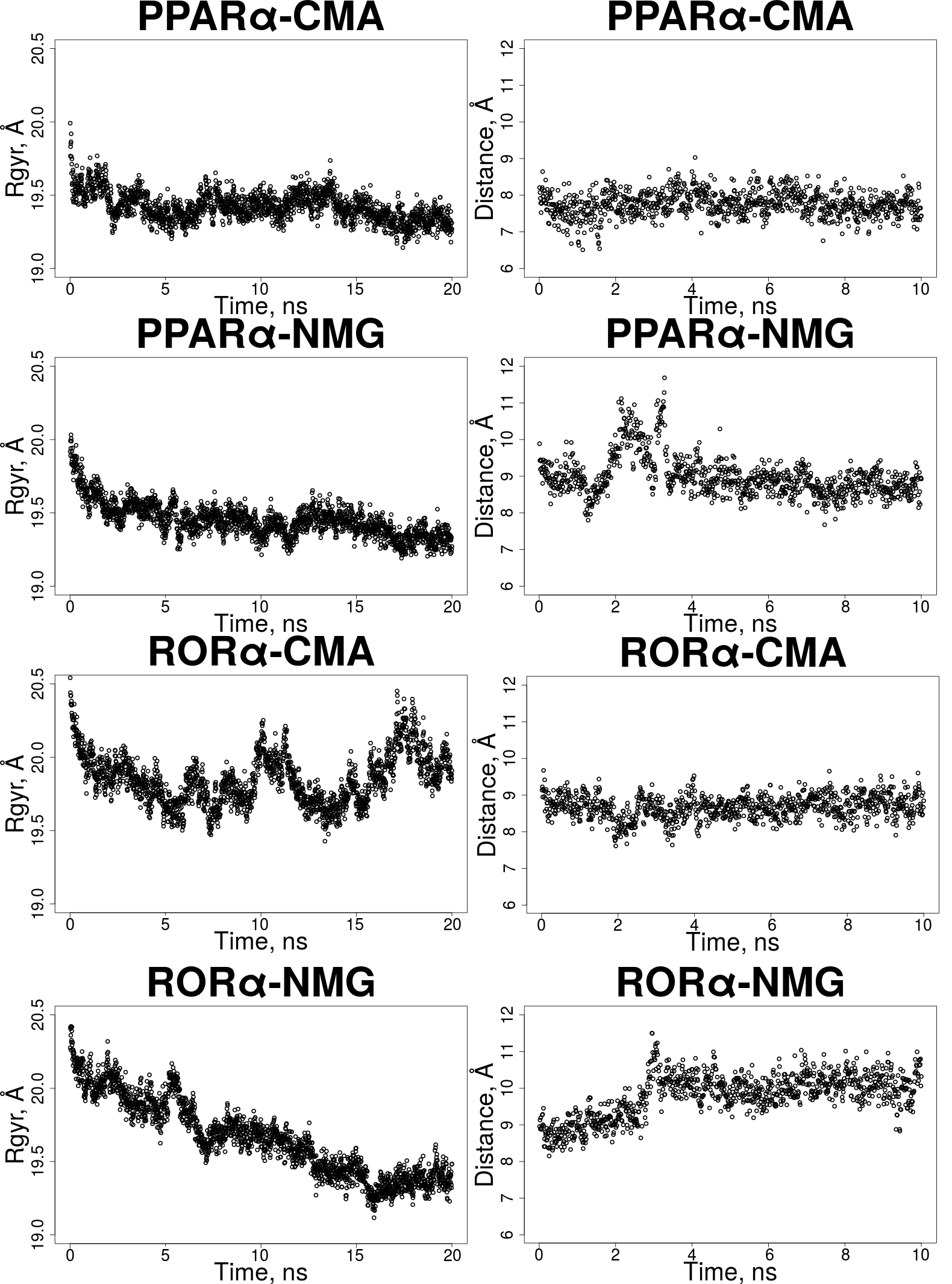
**

**Figure S4. MD simulations corresponding to the lowest binding energies for CMA and N-methylglucamine (abbreviated as NMG) in complexes with PPA**Rα **and/or RO**Rα **receptors: change of ligand conformation.** Proteins are shown in cartoon representation and ligands are represented as sticks. Colors correspond to snapshots (5 ns, 10 ns, 15 ns, 20 ns) and change from red to blue.


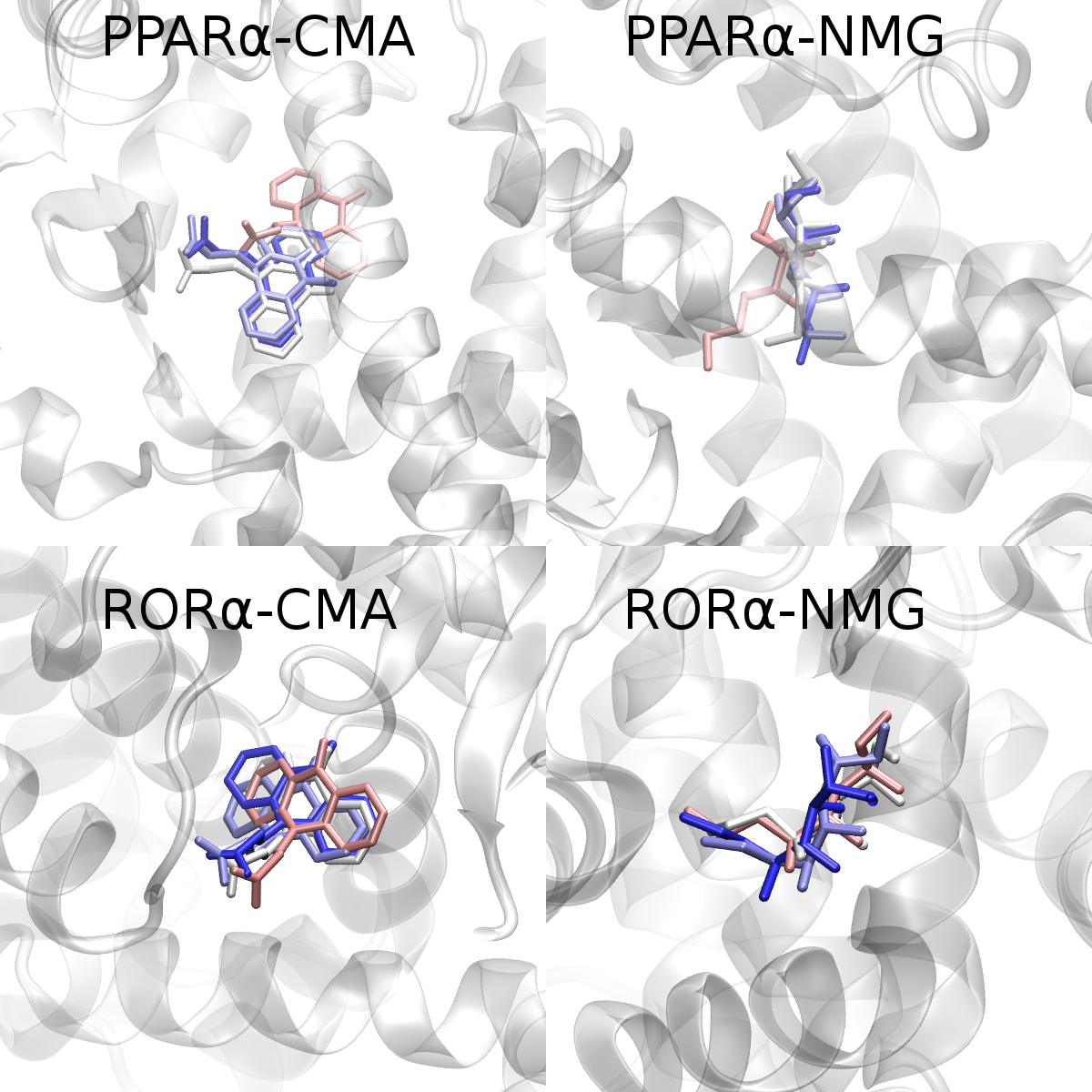

Supplement: Supplementary file 1 — Supplementary information [file 41598_2019_54208_MOESM1_ESM.doc]
